# Constructing Co_3_S_4_ Nanosheets Coating N‐Doped Carbon Nanofibers as Freestanding Sulfur Host for High‐Performance Lithium–Sulfur Batteries

**DOI:** 10.1002/advs.202002037

**Published:** 2020-10-11

**Authors:** Xuzi Zhang, Chaoqun Shang, Eser Metin Akinoglu, Xin Wang, Guofu Zhou

**Affiliations:** ^1^ Guangdong Provincial Key Laboratory of Optical Information Materials and Technology & Institute of Electronic Paper Displays South China Academy of Advanced Optoelectronics South China Normal University Guangzhou 510006 China; ^2^ International Academy of Optoelectronics at Zhaoqing South China Normal University Zhaoqing 526060 China

**Keywords:** catalytic, Co_3_S_4_ nanosheets, fast conversion, freestanding carbon nanofibers, high sulfur loading

## Abstract

Lithium–sulfur batteries (LSBs) have shown great potential as a rival for next generation batteries, for its relatively high theoretical capacity and eco‐friendly properties. Nevertheless, blocked by the shuttle effect of lithium polysulfides (LPSs, Li_2_S_4_‐Li_2_S_8_) and insulation of sulfur, LSBs show rapid capacity loss and cannot achieve the practical application. Herein, a composite of carbon nanofibers coated by Co_3_S_4_ nanosheets (denoted as CNF@Co_3_S_4_) is successfully synthesized as freestanding sulfur host to optimize the interaction with sulfur species. The combination of the two materials can lead extraordinary cycling and rate performance by alleviating the shuttle of LPSs effectively. N‐doped carbon nanofibers serve as long‐range conductive networks and Co_3_S_4_ nanosheets can accelerate the conversion of LPSs through its electrocatalytic and chemical adsorption ability. Benefiting from the unique structure, the transporting rate of Li^+^ can be enhanced. Distribution of Li^+^ is uniform for enough exposed negative active sites. As a result, the cell with CNF@Co_3_S_4_ as sulfur host is able to stabilize at 710 mA h g^−1^ at 1 C after 200 cycles with average coulombic efficiency of 97.8% in a sulfur loading of 1.7 mg cm^−2^ and deliver 4.1 mA h cm^−2^ at 0.1 C even in 6.8 mg cm^−2^ for 100 cycles.

## Graphical abstract

CNF@Co_3_S_4_ as a freestanding sulfur host optimizes the interaction between sulfur species and the host material by the polarity and catalytical effect of Co_3_S_4_ nanosheets. The electronegative property of Co_3_S_4_ can further alleviate the growth of Li‐dendrites through realizing the uniform Li^+^ flux and deposition. These advantages guarantee extraordinary cycling and rate performance of corresponding Li–S batteries.

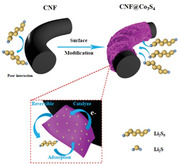

With the increasing development of energy storage field, batteries with much higher energy density and lower costs are urgently needed to satisfy the rising demands of portable devices and electrical vehicles.^[^ ^1^ ^]^ Lithium–sulfur batteries (LSBs) have come gradually into people's perspective for its high theoretical specific capacity (1675 mA h g^−1^) and energy density (2600 W h kg^−1^).^[^ ^2^ ^]^ Beyond that, the lower price and abundant natural reserves of sulfur also benefit the future practical of LSBs.^[^ ^3^ ^]^ However, the research of LSBs is lagged by the “shuttle effect” caused by the free‐diffusion of lithium polysulfides (LPSs, Li_2_S_4_‐Li_2_S_8_)^[^ ^4^ ^]^ and the insulation of sulfur. These follow continuous capacity loss and poor cyclic stability, which obstructs the practical application of LSBs.

To address the issues above, extensive efforts and strategies have been made, including designing high‐performance sulfur host materials,^[^ ^5^ ^]^ modifying the separator^[^ ^6^ ^]^ and developing novel electrolyte additives.^[^ ^7^ ^]^ In particular, designing proper host materials for sulfur is a more direct way. The traditional idea is to disperse the sulfur in conductive carbonaceous materials, such as carbon nanotube,^[^ ^8^ ^]^ graphene,^[^ ^9^ ^]^ hollow carbon nanosphere^[^ ^10^ ^]^ and carbon nanofibers.^[^ ^11^ ^]^ However, with the nonpolar surface, carbonaceous materials, only possessing physical adsorption, have weak chemical interaction with LPSs,^[^ ^12^ ^]^ which would result in poor cycling performance of LSBs. To enhance the chemisorption with LPSs, polar materials with good electric performance (e.g., MXene,^[^ ^13^ ^]^ TiN,^[^ ^14^ ^]^ MoS_2_,^[^ ^15^ ^]^ Ti_4_O_7_,^[^ ^16^ ^]^ CoS_2_,^[^ ^17^ ^]^ and Co_3_S_4_^[^ ^18^ ^]^) have been combined with carbonaceous materials as a sulfur host, which is also a proper way to optimize the conductivity of sulfur and Li_2_S. Among the diverse materials, cobalt sulfides become the strong competitors for its strong polarity and better electrical conductivity.^[^ ^18^, ^19^ ^]^ Pu et al.^[^ ^18^ ^]^synthesized Co_3_S_4_ nanotube as sulfur host, which reached a specific capacity of 517 mA h g^−1^ at 5 C and maintained 305 mA h g^−1^ after 1000 cycles. Chen et al.^[^ ^20^ ^]^ and Zhang et al.^[^ ^21^ ^]^ fabricated Co_3_S_4_ nanoboxes through ZIF‐67 combined with CNT as sulfur host, which showed initial specific capacities of 965 and 954 mA h g^−1^ at 1 C, respectively. In those reports, Co_3_S_4_ serves as the vital role for adsorbing LPS and catalyzing its conversion. Introducing heteroatom‐doped sites (such as N,^[^ ^22^ ^]^ P,^[^ ^10a^ ^]^ S,^[^ ^23^ ^]^ and B^[^ ^24^ ^]^) in carbonaceous scaffolds is another functionalized way, which can be also conductive to the chemisorption with LPSs caused by the charge rearrangement between carbon atom and heteroatoms. Heteroatom doping in scaffolds mitigates the diffusion of LPSs via strong chemical adsorption and is favorable for high utilization of sulfur for LSBs.

To fulfill the requirements mentioned above, here we propose a novel composite with 3D N‐doped carbon nanofibers coated by 2D Co_3_S_4_ nanosheets (CNF@Co_3_S_4_) serving as an effective sulfur host. Owning strong polarity and attractive room‐temperature conductivity of 3.3 × 10^3^ S cm^−1^,^[^ ^21^ ^]^ Co_3_S_4_ nanosheets can effectively realize the chemisorption of LPSs and fast conversion between LPSs and Li_2_S. In addition, N‐doped carbon nanofibers not only provide more polar sites and long electron conduction ways, but also serve as a free‐standing substrate, eliminating the invalid weight (e.g., Al foil and binder). Furthermore, the large specific area moderates effectively the volume expansion of sulfur. With the integration of 3D CNF and 2D Co_3_S_4_ nanosheets, LSBs can achieve stable cycling performance, remarkable rate performance and excellent areal capacity of 4.1 mA h cm^−2^ at 0.1 C with a high sulfur loading of 6.8 mg cm^−2^ for 100 cycles.

**Figure** **1** shows the schematic illustration about the synthetic process of self‐supporting carbon nanofibers coated by Co_3_S_4_ nanosheets (CNF@Co_3_S_4_). First, 3D carbon nanofibers (CNF), prepared by electrospinning method, are used as the long‐range conductive substrate. CNF are interwoven together to form a freestanding structure, which can provide enough physical barrier to block the diffusion of LPSs. Then 2D Co_3_S_4_ nanosheets are grown on the surface of carbon nanofibers to form a coating layer through hydrothermal method, which can enhance the interaction with LPSs for the increasing active area. Owning to the polarity and catalytic effect of Co_3_S_4_, shuttle effect of LPSs is hindered effectively when CNF@Co_3_S_4_ is used as sulfur host when cycling. In contrast, the soluble LPSs intermediately shuttle to the lithium surface with CNF as the sulfur host, leading to continuous loss of active sulfur and corrosion of lithium.

**Figure 1:**
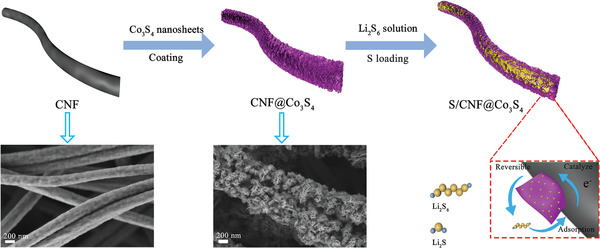
Schematic illustration of the process about synthesizing S/CNF@Co_3_S_4_ and the interaction between Li_2_S_6_ and Co_3_S_4_ nanosheets.

The morphology and structure of CNF@Co_3_S_4_ are investigated by scanning electron microscopy (SEM) and transmission electron microscopy (TEM). As shown in **Figure** **2**a,b, the CNF are interwoven together, resulting in a 3D freestanding structure with abundant Co_3_S_4_ nanosheets growing on the surface. The more structural details about CNF@Co_3_S_4_ are shown in TEM images. As shown in Figure 2c, the surface of CNF@Co_3_S_4_ is rough with more active sites. In the high‐resolution TEM image (Figure 2d), a well‐resolved lattice spacing of 0.298 nm is observed clearly and is agreed well with (311) planes of Co_3_S_4_. The selected area electron diffraction (SAED, inset of Figure 2d) pattern further indicated the polycrystalline type of Co_3_S_4_. In addition, the corresponding elemental maps of Co, S, and N show that cobalt and sulfur are distributed uniformly along the CNF, which means the successful growth of Co_3_S_4_ on the surface of CNF. As compared in Figure S1 (Supporting Information), the surface of CNF is smooth with diameter about 360 nm, while the diameter of CNF@Co_3_S_4_ is about 700 nm, indicating the thickness of Co_3_S_4_ is ≈170 nm. Without the coarse surface as CNF@Co_3_S_4_, CNF can't afford enough active sites for positive interaction with LPSs. Particularly, plenty small‐sized nanosheets of CNF@Co_3_S_4_ can further provide more storage space for electrolyte penetration and then ensure the fast diffusion of Li^+^. To further investigated the structural and ingredient details of CNF@Co_3_S_4_, BET and Raman are performed. As shown in Figure S3a (Supporting Information), CNF coated by Co_3_S_4_ nanosheets have a high BET (Brunauer–Emmett–Teller) specific surface area of 332 m^2^ g^−1^, while the CNF are measured at 290 m^2^ g^−1^. Meanwhile, the pore size distribution (inset of Figure S2a, Supporting Information) indicates the microporous structure of CNF@Co_3_S_4_ which is mainly centered at 1–3 nm. The high specific surface area can afford more anchoring sites for the adsorption of polysulfides and accelerate the Li^+^ diffusion, which may make for the high loading of sulfur. Raman spectra shown in Figure S2b (Supporting Information) indicates the nearly unchanged degree of graphitization after coating the layer of Co_3_S_4_ nanosheets on the carbon nanofibers for the small difference value of *I*_D_/*I*_G_ between CNF@Co_3_S_4_ and CNF. And the small peak appearing at about 667 cm^−1^ indicates the characteristic peak of Co_3_S_4_. The specific molar ratio of element Co and S is calculated by inductively coupled plasma atomic emission spectrometer (ICP). The result shown in Table S1 (Supporting Information) indicates that the molar ratio of Co and S is close to 3:4, which further confirms the successful synthesis of Co_3_S_4_ nanosheets.

**Figure 2:**
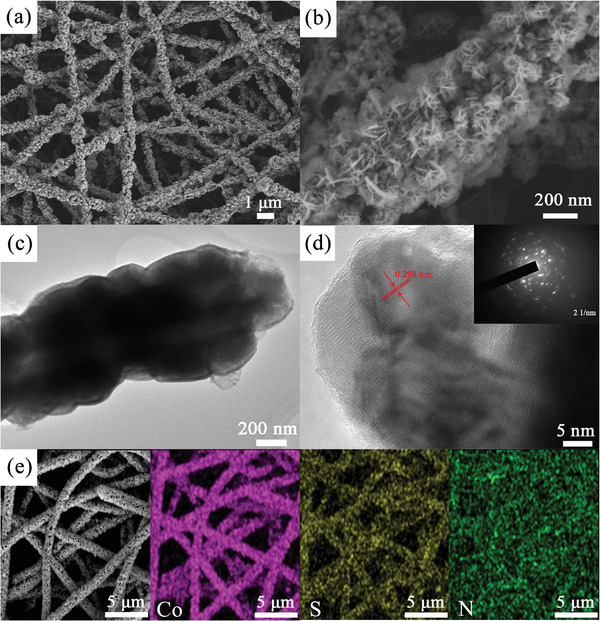
a,b) SEM images, c,d) TEM images and e) EDS mapping of CNF@Co_3_S_4_.

To directly investigate the chemical interaction of polysulfides with CNF@Co_3_S_4_, Li_2_S_6_ was employed as the representative polysulfide species to perform the adsorption measurements and X‐ray photoelectron spectroscopy (XPS) was applied to analyze the chemical interaction between polysulfides and CNF@Co_3_S_4_. Compared with CNF, the color of CNF@Co_3_S_4_ with Li_2_S_6_ solution (**Figure** **3**a) starts to change from yellow to colorless after 24 h, which can be ascribed to the strong polarity of Co_3_S_4_ nanosheets and the larger specific surface area than those of CNF. In addition, Figure 3b shows the UV–vis spectra of the three liquid supernatants after adsorption for 24 h. The peak at about 425 nm can be assigned to S_4_^2−^, and the peaks at low wavelength to the S_6_^2−^ and S_8_^2−^,^[^ ^25^ ^]^ which further confirm the adsorption of LPSs. Meanwhile, the remaining solid, named CNF@Co_3_S_4_‐Li_2_S_6_, is collected after drying in argon atmosphere and subjected to XPS characterization (Figure S3, Supporting Information). As shown in Figure 3c, the XPS Co 2p spectra of pristine CNF@Co_3_S_4_ shows the typical Co 2p_3/2_ and Co 2p_1/2_ spin‐orbit doublets and their associated shakeup satellites (identified as “Sat.”). Both doubles can be deconvoluted into two peaks, with peaks at 798.65 and 782.70 eV are attached to Co^2+^, while 797.20 and 781.06 eV are to Co^3+^.^[^ ^26^ ^]^ For CNF@Co_3_S_4_‐Li_2_S_6_, the peaks of both Co 2p_1/2_ and Co 2p_3/2_ shift to lower binding energy and the most obvious variation happen in peaks of Co^3+^ for the relative intensity weaker than CNF@Co_3_S_4_, which means the electron transporting mainly from S anion to Co^3+^ and further indicates that Co_3_S_4_ nanosheets can adsorb many polysulfides on its surface. As for N 1S spectra (Figure 3d), Li—N bond and S‐N appear at 407.53 and 399.38 eV for CNF@Co_3_S_4_‐Li_2_S_6_,^[^ ^21^ ^]^ while CNF@Co_3_S_4_ indicates typical peaks of nitrogen, graphitic‐N (400.90 eV), pyrrolic‐N (399.75 eV) and pyridinic‐N (398.25 eV). The two evident Li—N and S—N bonds confirm the interaction between N‐doped sites in carbon nanofibers and Li_2_S_6_. Furthermore, the existence of Co_3_S_4_ can promote the formation of Li_2_S. It is reported that the content ratio of Li_2_S_2_/Li_2_S could be estimated by the peak area ratio of S_T_^−1^ (Li_2_S_2_) and S^−2^ (Li_2_S) in S 2p spectra (Figure S4, Supporting Information).^[^ ^27^ ^]^ As shown in Figure S4 (Supporting Information), after the first discharge at 0.1 C, the ratio of S^2−^/S_T_^1−^ on the surface of CNF@Co_3_S_4_ is 1.87, which is much higher than that of CNF (0.72), indicating high Li_2_S generation with Co_3_S_4_ to reduce LPSs during discharge.

**Figure 3:**
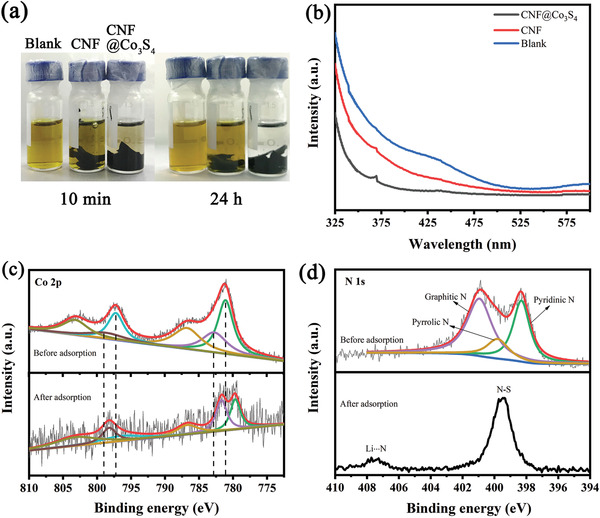
a) Image of the Li_2_S_6_ adsorption test and b) UV–vis spectra for Blank Li_2_S_6_, CNF and CNF@Co_3_S_4_. c) XPS of Co 2p peak and d) N 1s peak.

To further investigate the electrochemical performance of CNF@Co_3_S_4_, coin cells were assembled for LSBs. Particularly, the different sulfur loading can be achieved by changing the amount of Li_2_S_6_ catholyte dropped. The electrochemical behaviors are detected via cyclic voltammetry (CV), rate test and galvanostatic charge/discharge test. The CV curves of CNF@Co_3_S_4_ compared with CNF are shown in **Figure** **4**a. Both of the two curves contain two typical cathodic peaks located at ≈2.0 and ≈2.3 V, which are ascribed to transformation of sulfur molecule to long – chain polysulfides and subsequent reduction to solid Li_2_S_2_/Li_2_S. In the oxidation process, the broad anodic peak splits into two small peaks which are originated from Li_2_S to LPSs and then LPSs to sulfur. It's clear that the peaks of cell with CNF@Co_3_S_4_ are much higher and evident than that of CNF, indicating the more completed oxidation and reduction of sulfur species. Furthermore, in the cathodic process, the cell with CNF@Co_3_S_4_ displays higher potential relative to CNF, which means a lower polarization and faster kinetics in the transformation of LPSs.^[^ ^28^ ^]^ The fast reaction kinetics can also be deduced from the Tafel plots (Figure S5d, Supporting Information), where the cell with CNF@Co_3_S_4_ shows the lower Tafel slope.^[^ ^29^ ^]^ In the galvanostatic charge/discharge study at 0.2 C (Figure S5a, Supporting Information), the cell with CNF@Co_3_S_4_ shows remarkable performance for the lower ΔE (the over‐potential between the charge and discharge plateaus) of 0.17 V than 0.30 V of CNF with the assistance of Co_3_S_4_ nanosheets and higher specific capacity of 1120 mA h g^−1^. The rate performance is shown in Figure 4b. the cell with CNF@Co_3_S_4_ gives higher specific capacities of 1079, 1034, 970, 905, and 850 mA h g^−1^ at 0.1, 0.2, 0.5, 1, and 1.5 C, respectively. Even when the current density is increased to 1.5 C, the charge/discharge profiles of CNF@Co_3_S_4_ are still retained (Figure S5b, Supporting Information), suggesting an excellent rate performance, while CNF shows poor electrochemical performance without the aid of Co_3_S_4_ (Figure S5c, Supporting Information).

**Figure 4:**
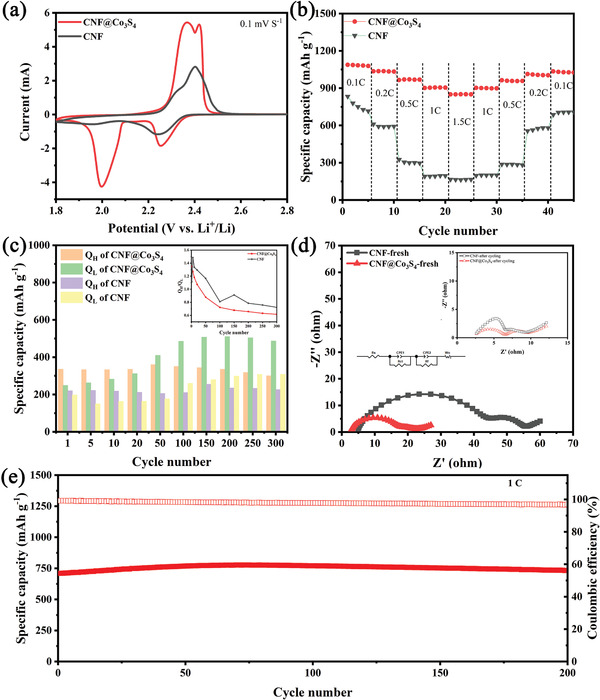
Electrochemical tests of LSBs with S/CNF@Co_3_S_4_ compared with S/CNF. a) CV curves at a scan rate of 0.1 mV s^−1^. b) Rate performance. c) The capacities of high (*Q*_H_) and low (*Q*_L_) plateau at different cycle at 0.5 C (Inset: The value of *Q*_H_/*Q*_L_ of CNF@Co_3_S_4_ and CNF). d) Electrochemical impedance spectra before and after cycling under open‐circuit conditions. e) Long‐cycle test of cells with S/CNF@Co_3_S_4_ at 1 C for 200 cycles. (All the tests above adapt a sulfur loading of 1.7 mg cm^−2^.)

The interaction between sulfur species and CNF@Co_3_S_4_ can be further evaluated by comparing the value of *Q*_H_/*Q*_L_ with different cycles (*Q*_H_ and *Q*_L_ represent the discharge capacity at high and low plateau, respectively). As shown in Figure 4c, the cell with CNF@Co_3_S_4_ as the freestanding sulfur host can deliver higher *Q*_H_ and *Q*_L_ than those of CNF, indicating that CNF@Co_3_S_4_ can block and utilize LPSs in cathodic side effectively. Furthermore, the ratio of *Q*_H_/*Q*_L_ (inset of Figure 4c) is related to the transformation of LPSs to Li_2_S_2_/Li_2_S. The lower *Q*_H_/*Q*_L_ of the cell with CNF@Co_3_S_4_ suggests that the conductive and polar Co_3_S_4_ nanosheets can promote the reduction of LPSs.

EIS was further performed to reveal the effect of Co_3_S_4_ on the kinetic behavior. As shown in Figure 4d, the impedance spectrum of the fresh cell is composed of two connective semicircles in high‐frequency region and an inclined line in low‐frequency region. The equivalent circuit after fitting is shown as an inset. The intercept on the axis represents the ohmic resistance (*R*_s_) related to the viscosity and chemical composition of electrolyte. The semicircle in the high frequency is ascribed to the charge‐transfer resistance (*R*_ct_) while the other semicircle in the middle‐frequency is attributed to the formation of Li_2_S_2_/Li_2_S (*R*_f_).^[^ ^30^ ^]^ The inclined line is attributed with the diffusion impedance (*W*_o_) of Li^+^. The fitting results have been listed in Table S2 (Supporting Information). Obviously, all the impedances of CNF@Co_3_S_4_ are lower than those of CNF, indicating the enhanced conductivity and accelerated Li^+^ diffusion. The EIS after 20 cycles is shown inset of Figure 4d. The values of *R*_ct_ and *R*_f_ decreased dramatically for the redistribution of sulfur species and rendering more exposed surface. In addition, *R*_ct_ and *R*_f_ of the cell with CNF@Co_3_S_4_ are 4.2 and 3.6 Ω, respectively, which are still lower than those of CNF (Table S2, Supporting Information).

The long‐cycling stability of the two materials is also evaluated. As shown in Figure S5e (Supporting Information), the specific capacity with the CNF@Co_3_S_4_ as the freestanding sulfur host can reach 789 mA h g^−1^ at 0.5 C after 300 cycles, while that of CNF is only 534 mA h g^−1^ after 300 cycles. In addition, the obvious increasing capacity in the first few dozen cycles can be attributed to the redistribution of LPSs on the surface and in the inner space of CNF@Co_3_S_4_, indicating an improved utilization of sulfur species. When the current density increases up to 1 C (Figure 4e), LSBs with CNF@Co_3_S_4_ can deliver an initial specific capacity of 710 mA h g^−1^ and an ultrastable cycling stability with the capacity retention nearly 100% and a high average coulombic efficiency of 97.8% after 200 cycles. The excellent cycling performance are superior to other transition metal compound‐based sulfur immobilizers (Table S4, Supporting Information). Additionally, samples with different hydrothermal temperature as illustrated in Figure S6 (Supporting Information) demonstrate that the appropriate growth of Co_3_S_4_ plays important role in the efficient utilization of S species.

The catalytic and kinetic effect of 2D Co_3_S_4_ nanosheets on the LPSs redox reaction was further explored by symmetric batteries. **Figure** **5**a shows the CV curves of CNF@Co_3_S_4_ and CNF as the symmetric electrodes at 0.5 mV s^−1^ in the voltage window from −0.8 to 0.8 V. Without Li_2_S_6_, CNF@Co_3_S_4_ exhibits no reactivity in the electrolyte. After adding Li_2_S_6_, two pairs of distinct peaks corresponding to the oxidation and reduction of LPSs are observed in CNF@Co_3_S_4_, where peaks of a and b are ascribed to the reduction of Li_2_S_6_/Li_2_S_8_ to Li_2_S and peaks of c and d are attributed to the oxidation of Li_2_S to Li_2_S_6_/Li_2_S_8_.^[^ ^31^ ^]^ However, CNF shows only two broad peaks and lower response current, which means the weak catalytic activity on the oxidation and reduction of LPSs. Apart from the tests mentioned above, the Li_2_S precipitation kinetics of LPSs on the surface of CNF@Co_3_S_4_ and CNF were monitored by and potentiostatically discharge with Li_2_S_8_ catholyte as the active material at 112 µA and 2.10 V.^[^ ^32^ ^]^ As shown in Figure 5b, after potentialstastic discharge for ≈2 h, the currents for both cells reach the peak top, which indicates the deposition of Li_2_S on the surface of CNF@Co_3_S_4_ and CNF. The cell with CNF@Co_3_S_4_ as the freestanding sulfur host shows a larger deposition capacity of 1.25 mA h than 1.14 mA h of CNF, reflecting the more efficient utilization of LPSs caused by the trapping and catalytic effect of the Co_3_S_4_ nanosheets.

**Figure 5:**
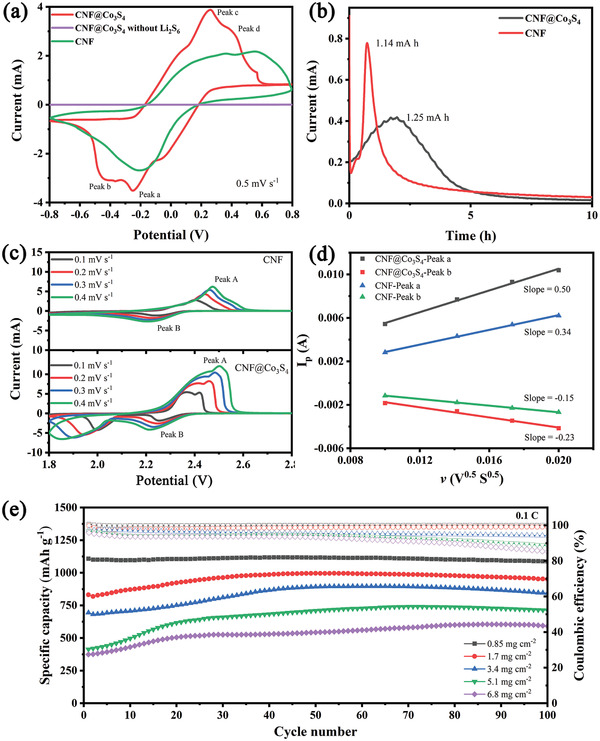
a) CV of symmetric cells at 0.5 mV s^−1^. b) Potentiostatic discharge curves with Li_2_S_8_ catholyte. c) CV curves of cells with CNF@Co_3_S_4_ and CNF at different scan rates and d) corresponding linear fits of the CV peak currents. e) Cycling performance of LSBs with different sulfur loading at 0.1 C.

To deeply understand the effect of CNF@Co_3_S_4_ for the better potential polarization performance, CV with different scan rate ranging from 0.1–0.4 mV s^−1^ compared with cells using CNF as the sulfur host was conducted (Figure 5c). Figure 5d exhibits the fitting curves of peak current of cells with CNF@Co_3_S_4_ and CNF on the basis of Randles–Sevick equation as following:^[^ ^33^ ^]^ *I*_p_ = 2.69 × 10^5^ *n* ^3/2^A$D_{\mathrm{Li}+}^{1/2}$ *v* ^1/2^ *C*_Li+_, in which *D*_Li+_ represents lithium ion diffusion coefficient (cm^2^ s^−1^), *I*_p_ stands for the peak current (A), *n* is the number of electrons involved in the reaction (*n* = 2 for LSBs), A is the area of electrode (cm^2^), *C*_Li+_ refers to the lithium ion concentration (mol L^−1^), and *v* represents the scanning rate (V s^−1^). The calculated values of *D*_Li+_ is shown in Table S3 (Supporting Information). The *D*_Li+_ of Peak A and B for cell with CNF@Co_3_S_4_ are 3.38 × 10^−13^ and 1.56 × 10^−13^ cm^2^ s^−1^, respectively, which are both higher than those with CNF (3.04 × 10^−14^ cm^2^ s^−1^ of peak A and 7.15 × 10^−14^ cm^2^ s^−1^ of peak B). This indicates that the CNF@Co_3_S_4_ can guarantee a rapid lithium ion transfer rate compared with CNF.

After cycling, the cells were disassembled after 40 cycles at 0.2 C with fully discharged state at the end. The surface morphology of the lithium foil of both cells are shown in Figure S7 (Supporting Information). The photo of separator and lithium foil (against separator) of cell with CNF@Co_3_S_4_ (Figure S7a, Supporting Information) exhibits paler yellow and less change than those of S/CNF, indicating few LPSs shuttling. Compared with the smooth surface morphology of the lithium foil used in the cell with CNF@Co_3_S_4_, the cell with CNF suffers from large cracks and severe pulverization of lithium foil (Figure S7c,d, Supporting Information). The improvement can be ascribed to the superior suppression for polysulfide diffusion by the CNF@Co_3_S_4_, which prevents the shuttle of soluble LPSs, thus leading to the protected morphology of lithium. Furthermore, it can be also explained through the more negative zeta potential of −27.78 mV of CNF@Co_3_S_4_ than −18.99 mV of CNF (Figure S7b, Supporting Information).^[^ ^30^ ^]^ The uniform coating of 2D Co_3_S_4_ nanosheets can optimize the Li‐ion flux through electrostatic interaction to reach a homogeneous diffusion and deposition on the surface of lithium foil, which efficiently prevent the formation of a large area of Li‐dendrites. To further examine the practical utilization of CNF@Co_3_S_4_ as the freestanding sulfur host, the mass loading of sulfur is raised from 0.85 to 6.8 mg cm ^−2^. As shown in Figure 5e, the cell with sulfur loading of 6.8 mg cm^−2^ shows high and stable specific capacity of 592 mA h g^−1^ (4.1 mA h cm^−2^ shown in Figure S8, Supporting Information) after 100 cycles at 0.1 C and high average coulombic efficiency of ≈94%, indicating the strong adsorption ability toward LPSs and highly catalytic effect of CNF@Co_3_S_4_.

In summary, we design a freestanding CNF@Co_3_S_4_ serving as an effective sulfur host. Owning to the polarity and catalytical effect of Co_3_S_4_ nanosheets, LPSs can be adsorbed effectively and achieve fast conversion to Li_2_S_2_/Li_2_S. The more electronegative property of Co_3_S_4_ nanosheets can further alleviate the growth of Li‐dendrites through realizing the uniform Li‐ion flux and deposition. As a result, LSBs with CNF@Co_3_S_4_ can achieve stable cycling performance at 0.5 C and 1 C, remarkable rate performance. Besides, the LSBs with CNF@Co_3_S_4_ also deliver excellent areal capacity of 4.1 mA h cm^−2^ at 0.1 C with a high sulfur loading of 6.8 mg cm^−2^ for 100 cycles.

## Experimental Section

##### Synthesis of the 3D N‐Doped Carbon Nanofibers

3D N‐doped carbon nanofibers was synthesized by electrospinning method following carbonization. In detail, 8 wt% polyacrylonitrile (PAN) solution with DMF as the solvent was prepared as the spinning solution. Before electrospinning, 20 mL of the spinning solution was poured into a 25 mL syringe. At surrounding temperature of 40 °C and air humidity of 55%, the pushing speed of syringe was set as 0.1 mm min^−1^, while a certain voltage applied between the needle and collector was 16 kV with a distance of 15 cm. After electrospinning process, the PAN fiber collected by the rotating aluminum foil was conducted to a continuous high‐temperature process, containing pre‐oxidation at 280 °C for 2 h in air and carbonization at 800 °C for 2 h in Ar with a heating rate of 2 °C min^−1^. Finally, 3D N‐doped carbon nanofibers (CNF) were obtained.

##### Synthesis of the CNF@Co_3_S_4_

CNF@Co_3_S_4_ was synthesized by a two‐step hydrothermal process. First, 30.00 mg CoCl_2_·6H_2_O, 46.30 mg NH_4_F, and 37.50 mg urea were dissolved in 50 mL deionized water. Few pieces of CNF with diameter of 12 mm were put into the resulting solution. Then the mixture was transferred to a 100 mL Teflon‐lined stainless‐steel autoclave and heated at 150 °C for 10 h. The obtained product was centrifugated and washed for several times with deionized water. Secondly, 0.12 mg Na_2_S·9H_2_O was dissolved in 50 mL of deionized water and then the obtained product was dropped into this solution. The resulting mixture was transferred into a 100 mL Teflon‐lined stainless‐steel autoclave and heated at 150 °C for 6 h. Finally, the CNF@Co_3_S_4_ was obtained.

##### Preparation of Li_2_S_6_ Catholyte and S/CNF@Co_3_S_4_

3.6 m Li_2_S_6_ was prepared by dissolving 1.66 g Li_2_S (Sigma‐Aldrich) and 5.76 g S (Macklin) in 10 mL electrolyte (1 m lithium bis(trifluoromethanesulfonyl)imide (LiTFSI) in 1,2‐dimethoxyethane (DME) and 1,3‐dioxolane (DOL) with volume ratio of 1:1 with the addition of 0.2 m LiNO_3_). Then the mixture was heated at 80 °C for 12 h under magnetic stirring. All this procedure above was required no water and no oxygen. 10 µL of 1.0 m Li_2_S_6_ (equivalent to 1.92 mg of sulfur loading) as the active material was dropped on the freestanding CNF@Co_3_S_4_ film to obtain the S composite (S/CNF@Co_3_S_4_). For comparison, S loading with different mass was realized by changing the volume of Li_2_S_6_ catholyte. The electrolyte/sulfur ratios are 15.6, 10.4, 8.7, and 7.8 mL g^−1^ for different sulfur loading of 1.7, 3.4, 5.1, and 6.8 mg cm^−2^, respectively. In addition, LSBs with CNF@Co_3_S_4_ needed to be activated with small current to diffuse the active material.

##### Characterization for CNF@Co_3_S_4_

The morphologies of the CNF@Co_3_S_4_ were characterized by scanning electron microscopy (ZEISS Ultra 55) and transmission electron microscopy (JEM‐2100HR). EDS mapping was also analyzed by EDAX analysis system of ZEISS Ultra 55. X‐ray photoelectron spectroscopy was gained from Thermo Scientific K‐Alpha with Al K*α* radiation of 30 eV. Brunner–Emmet–Teller measurements were recorded by Micromeritics TriStar II 3flex. Raman spectra was measured by Thermo Fischer DXR 2Xi. ICP test was conducted with Agilent 720ES.

##### Electrochemical Tests

LSBs were assembled in CR2032 coin cells with lithium metal as the reference electrode (diameter: 12 mm) and Celgard 2500 polypropylene membrane as the separator (diameter: 17 mm). The as‐prepared S/CNF@Co_3_S_4_ was served as the cathode (diameter: 12 mm). The amount of electrolyte (1 m LiTFSI in DME and DOL with volume ratio of 1:1 with the addition of 0.2 m LiNO_3_) used in the batteries is 20 µL. The concentration of LiNO_3_ is an important role to determine the performance of LSBs.^[^ ^34^ ^]^ LiNO_3_ can increase the utilization of sulfur species in lower concentration and help to protect the carbon matrix structure in higher concentration that 0.2 m is a moderate concentration to regulate these two competitive influences.

The above procedures were carried out in an Ar filled glove box (O_2_ < 0.1 ppm, H_2_O < 0.1 ppm). The cyclic voltammetry (CV) and electrochemical impedance spectroscopy (EIS) results were obtained from an electrochemical workstation (VMP3, Biologic) at a scan rate of 0.1 mV s^−1^ from 1.8 to 2.8 V. In addition, the galvanostatic discharge‐charge experiments were tested in a NEWARE battery testing system. The specific capacity was calculated with the mass of sulfur.

##### Symmetric Cell Assembly and Electrochemical Tests

For the symmetric electrochemical cells, the working electrode was CNF or CNF@Co_3_S_4_ with 10 µL of 1.0 m Li_2_S_6_. CV curves were measured at a scanning rate of 0.5 mV s^−1^ in a potential window from −0.8 to 0.8 V on a Biologic VMP3 electrochemistry workstation.

##### Measurement for Lithium Sulfide Nucleation

Li_2_S_8_ catholyte was applied as the active material and prepared by adding Li_2_S and S into the electrolyte of LSBs with a molar ratio of 1:7. CNF and CNF@Co_3_S_4_ were applied as the sulfur host while the lithium foil as the anode. 10 µL Li_2_S_8_ (1 m) catholyte was added into the sulfur host. The cell was first discharged at the current of 112 µA until the voltage reached 2.11 V, then held the voltage at 2.10 V until the current decreased to 10^−2^ mA.

## Conflict of Interest

The authors declare no conflict of interest.

## Supporting information

Supporting Information
