# Supplementary material for: Constructing Co3S4 Nanosheets Coating N‐Doped Carbon Nanofibers as Freestanding Sulfur Host for High‐Performance Lithium–Sulfur Batteries
Source: Adv Sci (Weinh). 2020 Oct 11;7(22):2002037. doi: 10.1002/advs.202002037 (PMC7675184; doi:10.1002/advs.202002037)
Supplement: Supplementary file 1 — Supporting Information [file ADVS-7-2002037-s001.pdf]

© 2020 Wiley-VCH GmbH

ADVANCED SCIENCE
Open Access

Supporting Information

for *Adv. Sci.,* DOI: 10.1002/advs.202002037

Constructing Co_3S_4 Nanosheets Coating

N-doped Carbon Nanofibers as Freestanding

Sulfur Host for High Performance Lithium-sulfur Batteries

Xuzi Zhang, Chaoqun Shang, Eser Metin Akinoglu,*
Xin Wang, and Guofu Zhou

Supporting information

Constructing Co_3S_4 nanosheets coating N-doped carbon nanofibers as freestanding sulfur host for high performance lithium-sulfur batteries

Xuzi Zhang, Chaoqun Shang, Eser Metin Akinoglu, Xin Wang, Guofu Zhou*

X. Zhang, Dr. C. Shang, Prof. X. Wang, Prof. G. Zhou
Guangdong Provincial Key Laboratory of Optical Information Materials and Technology & Institute of Electronic Paper Displays, South China Academy of Advanced Optoelectronics, South China Normal University, Guangzhou 510006, China
E-mail: chaoqun.shang@ecs-scnu.org
Dr. E. Akinoglu, Prof. X. Wang, Prof. G. Zhou
International Academy of Optoelectronics at Zhaoqing, South China Normal University, Zhaoqing 526060, China

Table S1 The content of Co and S derived from ICP test.

Element	Concentration （mg/L)	Mass content （mg/kg)	Molar ratio (Co/S)
Co	3.97	21334.1	0.79
S	2.70	14533.9	

Table S2 The fitting data of EIS spectra.

LSBs	R_s (Ω)	R_{ct} (Ω)	R_f (Ω)
CNF@Co_3S_4-fresh	2.4	13.3	14.0
CNF@Co_3S_4-after cycling	2.3	4.2	3.6
CNF-fresh	4.5	39.6	9.6
CNF-after cycling	3.6	6.6	4.2

Table S3 The calculated data of D_{Li+}

Peak	D_{Li+} (cm^2 s^{-1})
CNF@Co_3S_4-Peak A	3.38×10^{-13}
CNF@Co_3S_4-Peak B	1.56×10^{-13}
CNF-Peak A	3.04×10^{-14}
CNF-Peak B	7.15×10^{-14}

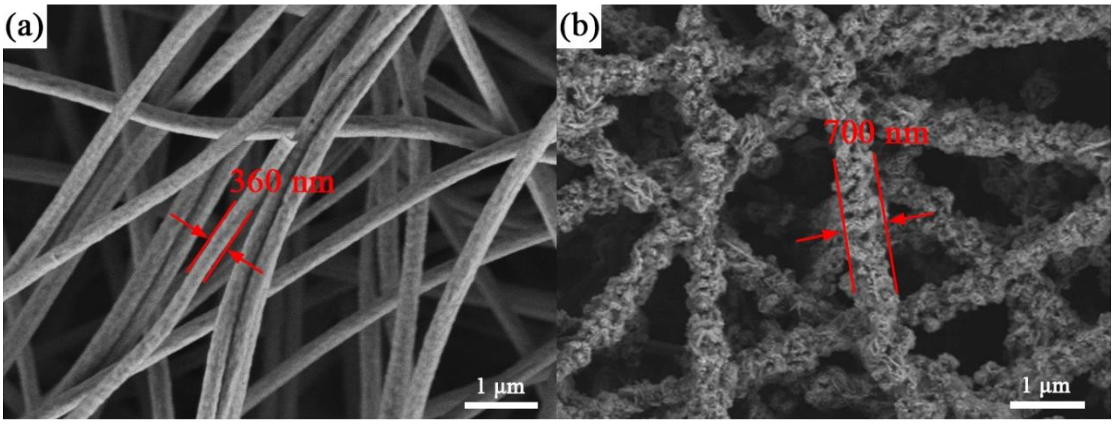

Figure S1 SEM of CNF (a) and CNF@Co_3S_4 (b).

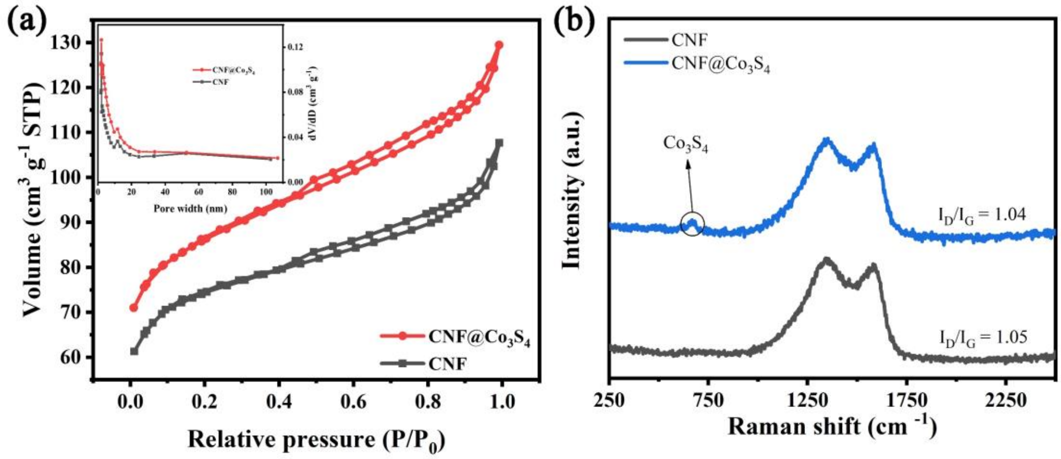

Figure S2 (a) N_2 adsorption/desorption isotherms and BJH pores distribution (inset). (b) Raman spectra.

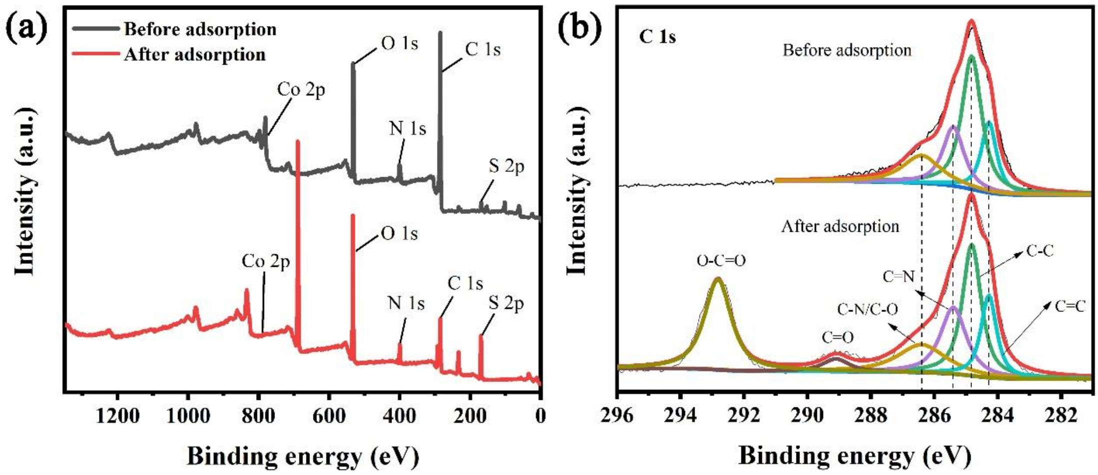

Figure S3 (a) XPS survey spectra of CNF@Co_3S_4 before and after adsorbing Li_2S_6.

(b) XPS of C 1s peak.

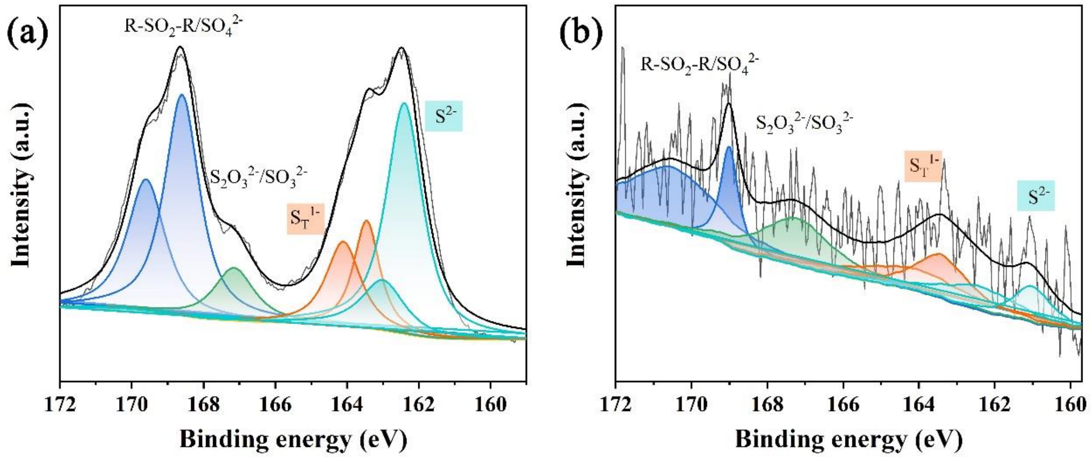

Figure S4 XPS analysis of S 2p in the discharge products on the surface of CNF@Co_3S_4 (a) and CNF (b) after the first discharge process.

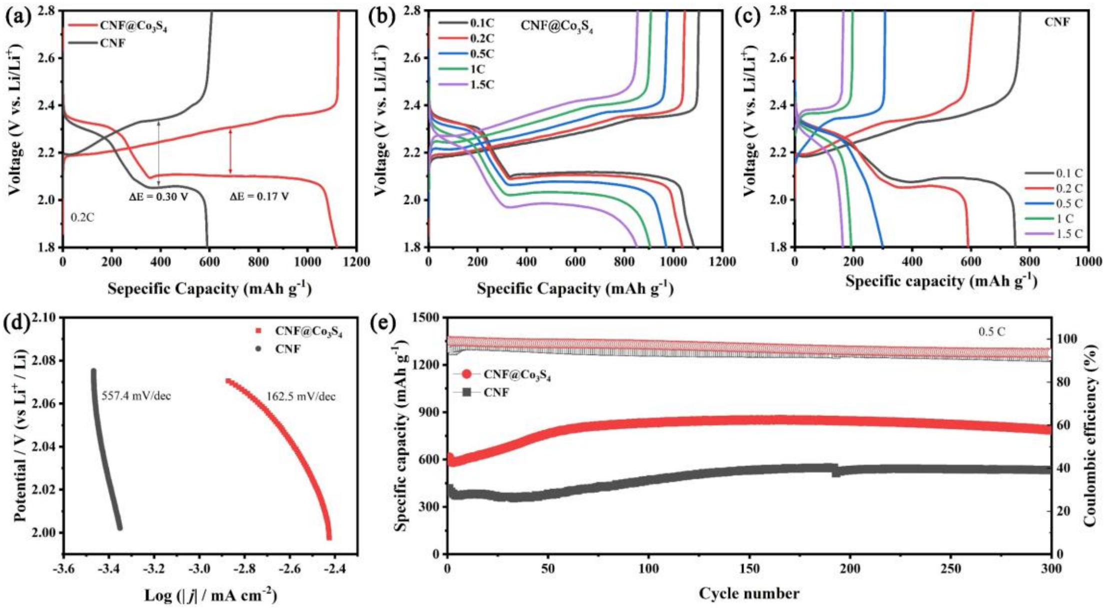

Figure S5 (a) Charge-discharge curves at 0.2C. Charge-discharge curves of CNF@Co_3S_4 (b) and CNF (c) with various scan rates. (d) Tafel plots derived from CV curves at 0.1 mV s^{-1}. (e) Cycling performance at 0.5C for 300 cycles.

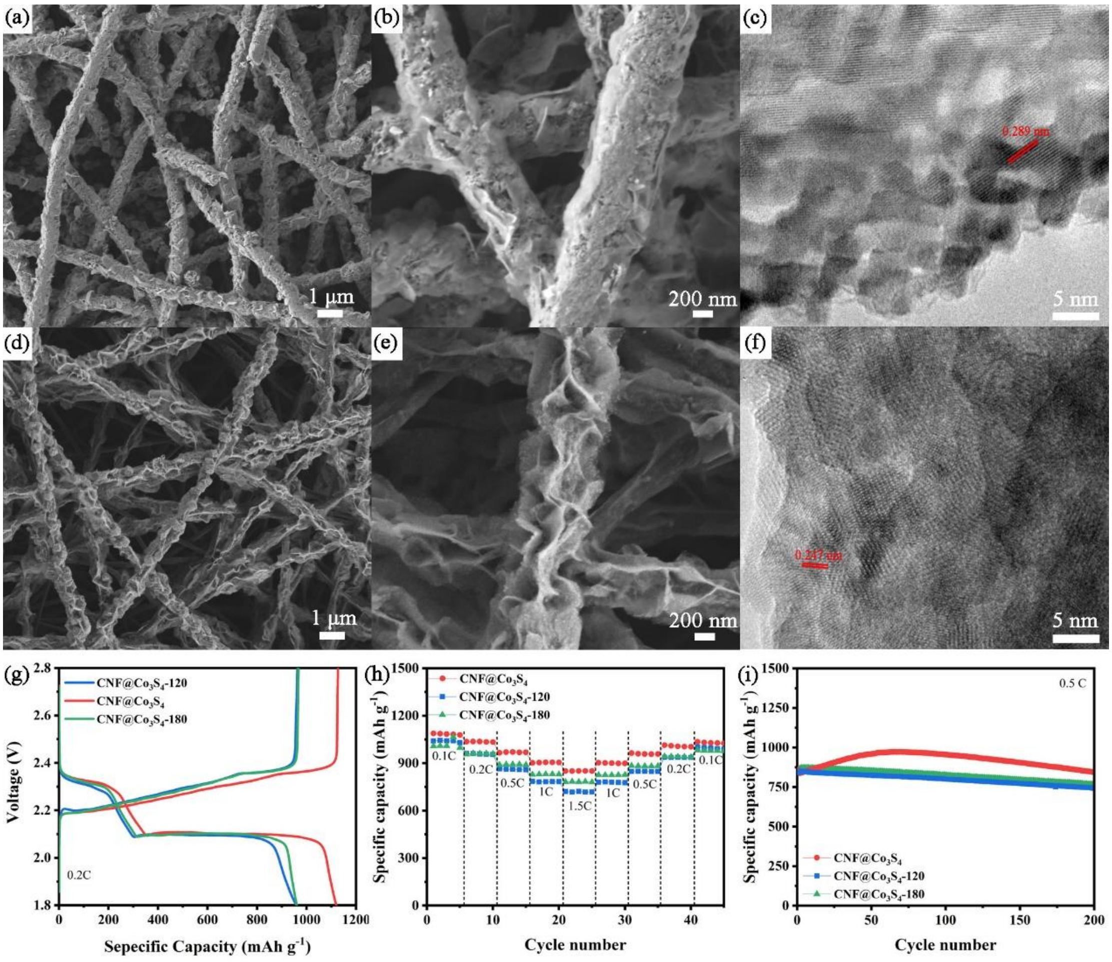

Figure S6 SEM and TEM of (a-c) CNF@Co_3S_4-120 and (d-e) CNF@Co_3S_4-180. (g) CV curves, (h) rate performance and (i) cycling performance of CNF@Co_3S_4 with different hydrothermal temperature.

For comparison, samples with different hydrothermal temperature are also evaluated. SEM of CNF@Co_3S_4 with different hydrothermal temperature of 120 °C and 180 °C (CNF@Co_3S_4-120 and CNF@Co_3S_4-180) are depicted in Figure S5. Compared with the CNF@Co_3S_4-120 (Figure S5a-c) and CNF@Co_3S_4-180 (Figure S5d-f), CNF@Co_3S_4 is coating more small-sized nanosheets, while can provide more active area for reaction of LPSs on the surface of Co_3S_4. Galvanostatic

charge-discharge curve at 0.2 C of LSBs with CNF@Co_3S_4 compared with those with CNF@Co_3S_4-120 and CNF@Co_3S_4-180 (Figure S5g) shows the largest specific capacity and lowest ΔE, further indicating that the nanosheets with much exposed surface can enhance the utilization of sulfur species and catalytic effect on transformation between CNF@Co_3S_4 and LPSs than bulk- and microsheet-Co_3S_4 grown on carbon nanofibers. Thanks to the largest specific surface area, CNF@Co_3S_4 is also performed the most excellent rate capability (Figure S5h) and cycling stability (Figure S5i).

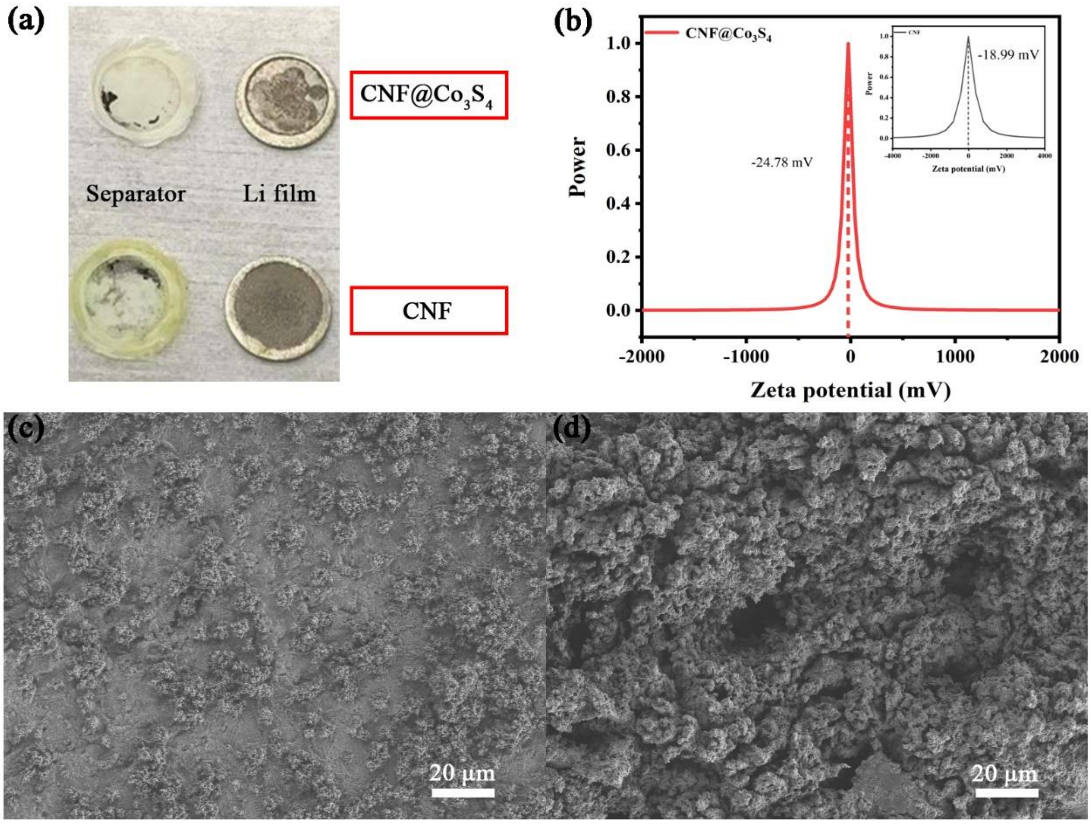

Figure S7 (a) Images of disassembled cells with S/CNFCo$_3$S$_4$ and S/CNF. (b) Zeta

potential of CNF@Co_3S_4 and CNF (inset). SEM images of lithium foil of cells with (c) S/CNFCo_3S_4 and (d) S/CNF.

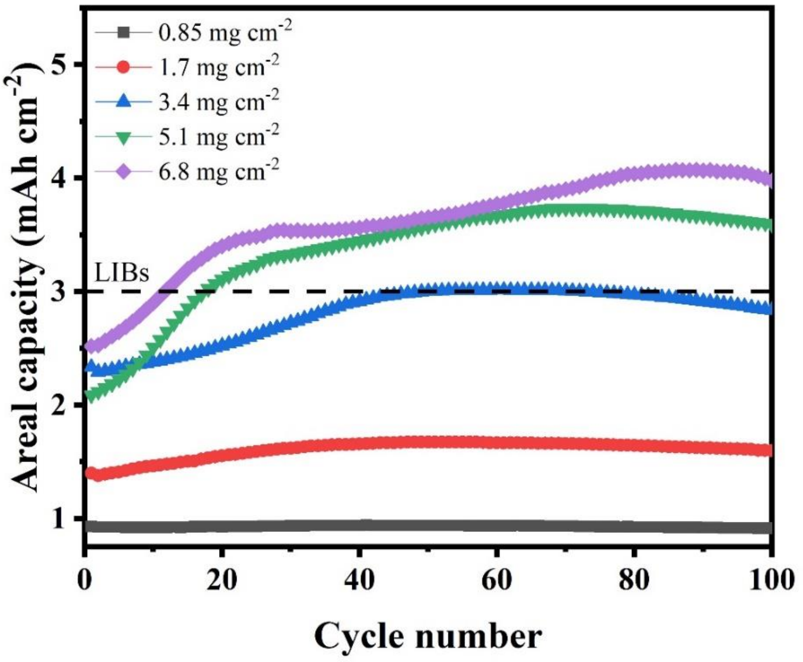

Figure S8 Areal capacity of LSBs for CNF@Co_3S_4 with different sulfur loading.

Table S4 Comparison of cycling performance in Li-S batteries with different transition metal compound based sulfur immobilizers.

Reference	Sulfur loading (mg cm^{-2})	Rate	Initial capacity (mA h g^{-1})	Capacity rentation	Materials
1	1.2	1 C	947.6	62.5 % (600 cycles)	Ultrathin MoS_2 Nanosheets
2	3.1-3.3	1 C	860.1	79 % (300 cycles)	$FeCo_2S_4$ Nanotube
3	2	0.5 C	1220	58.3 % (500 cycles)	TiO_2
4	1.1	1 C	~870	~85 % (150 cycles)	C@TiN nanosphere
5	2.3	0.5 C	724	96% (300 cycles)	NiS@C hollow spheres
This work	1.7	1 C	710	~100 % (200 cycles)	CNF@Co_3S_4

Reference:

[1] J. Wu, H. Zeng, X. Li, H. Pei, Z. Xue, Y.-S. Ye, X. Xie, *ACS Appl. Energy Mater.* 2019, **2**, 1702.

[2] B. Guo, S. Bandaru, C. Dai, H. Chen, Y. Zhang, Q. Xu, S. Bao, M. Chen, M. Xu, *ACS Appl. Mater. Interfaces* 2018, **10**, 43707.

[3] C. Zha, X. Gu, D. Wu, H. Chen, *J. Mater. Chem. A* 2019, **7**, 6431.

[4] Y. Wang, R. Zhang, Y.-c. Pang, X. Chen, J. Lang, J. Xu, C. Xiao, H. Li, K. Xi, S. Ding, *Energy Storage Mater.* 2019, **16**, 228.

[5] C. Ye, L. Zhang, C. Guo, D. Li, A. Vasileff, H. Wang, S.-Z. Qiao, *Adv. Funct. Mater.* 2017, **27**, 1702524.
